# Web-Based Eligibility Quizzes to Verify Opioid Use and County Residence Among Rural Young Adults: Eligibility Screening Results from a Feasibility Study

**DOI:** 10.2196/12984

**Published:** 2019-06-18

**Authors:** April M Ballard, Hannah LF Cooper, April M Young

**Affiliations:** 1 Department of Epidemiology University of Kentucky Lexington, KY United States; 2 Department of Environmental Health Emory University Rollins School of Public Health Atlanta, GA United States; 3 Department of Behavioral Sciences and Health Education Emory University Rollins School of Public Health Atlanta, GA United States; 4 Center on Drug and Alcohol Research University of Kentucky Lexington, KY United States

**Keywords:** Web-based methods, eligibility determination, rural health, substance-related disorders, opioid use, surveys and questionnaires, internet, confidentiality, sampling methods, recruitment

## Abstract

**Background:**

Web-based methods can be used to collect data from hidden populations, including people who use drugs (PWUD). These methods might be especially advantageous among PWUD in rural areas, where transportation barriers are prevalent, stigma may heighten concerns about confidentiality, and internet access is improving. However, Web-based research with PWUD can be challenging, especially in verifying eligibility. Administering *quizzes* to verify residential and substance use eligibility could prove valuable in online research among PWUD, yet the utility of this approach is currently unknown.

**Objective:**

This study describes the implementation of online eligibility quizzes about the local community to verify residence in the target study area along with drug dose, appearance, and price to verify opioid misuse.

**Methods:**

To be eligible, individuals had to live in 1 of 5 eastern Kentucky counties, report using opioids to get high in the past 30 days, and be 18 to 35 years old. Participants recruited from August 2017 to July 2018 were asked questions about their opioid use followed by a quiz about drug dose, appearance, and price to verify substance use eligibility. Residential eligibility was verified with 5-question quizzes assessing knowledge of the county where they reported living. Questions tested knowledge about towns, festivals, and landmarks; local school mascots and colors; and presence of certain retail stores, restaurants, and facilities (eg, jails). A subsample that reported using opioids in the past 24 hours was randomly selected to complete urine drug testing (UDT). Nonparametric tests were performed to explore differences across demographic subgroups.

**Results:**

Of the 410 entries assessed for eligibility, 39.3% (161/410) were ineligible as they reported no substance use, being outside the age range, or living outside the study area. Of the remaining 249 who met the eligibility criteria based on age, residency, and opioid misuse, 94.0% (234/249) *passed* the eligibility quizzes. Among those who passed the heroin quiz, 99.4% (167/168) recognized the image of powdered heroin, 94.6% (159/168) answered the cap size (ie, the purchase unit) question correctly, and 97.0% (163/168) answered the street price question correctly. Among those who passed the drug quiz for prescription opioids, 95% (36/38) answered the dose question correctly, and 82% (31/38) selected the correct image. In a random sample of participants who completed UDT within 3 days of their online screening, 74% (25/34) tested positive for an opioid.

**Conclusions:**

This study demonstrated the utility of using online eligibility screening quizzes to verify opioid misuse and residence. Participants accurately recognized heroin and prescription opioid doses, prices, and images and correctly answered questions about features of their county. Online quizzes to screen and enroll PWUD hold promise for future research as an alternative to more time- and resource-intensive approaches that could offset the advantages of Web-based methods.

## Introduction

### Background

Studies have shown that Web-based methods can successfully be used to collect data from hidden populations, including men who have sex with men (MSM), people with sexually transmitted infections, and people who use drugs (PWUD) [1-9]. Web-based data collection can decrease barriers to study participation by allowing individuals to complete surveys from any location and by providing participants with a heightened sense of anonymity [10-14]. These methods may be particularly pertinent for research among PWUD because of concerns around legality and stigma of the behaviors they are reporting. Furthermore, the importance of innovations in substance use research is heightened by the increase in substance use and related harms such as overdose mortality in several countries including the United States, Australia, and Europe [15,16].

Rural areas such as central Appalachia have a longstanding and continued problem with prescription opioid misuse and drug-related harms [17]. A steady increase in substance use has occurred over the past 2 decades, with abuse rates exceeding national averages [17]. Methodological innovations in research among rural US populations have, therefore, become increasingly important because of the disproportionate burden of opioid use and related harms (ie, hepatitis C and overdose) affecting rural young adults [17-23]. Furthermore, with 78% of rural adult Americans reporting use of the internet [24], the methodological advantages of Web-based data collection and recruitment might be especially advantageous for research among hard-to-reach populations in rural settings, where transportation barriers are prevalent and fear of breaches to confidentiality may be heightened because of stigma [25,26]. However, no studies to our knowledge have developed and piloted Web-based methods for data collection among rural PWUD, though a number of studies have used Web-based methods to recruit and collect data from PWUD [1,3,9,27-31] in urban settings.

Regardless of rurality, verifying eligibility criteria for studies related to recent substance use with PWUD and residence in a targeted geographic area can be challenging. Web-based methods can further compound these challenges. Substance use self-report can be employed for eligibility screening; however, validation studies using biologic techniques have shown a range of accuracy and under-reporting, with frequency and magnitude depending on drug class and socioecological factors [32,33]. Many in-person studies of PWUD use urine drug testing (UDT), saliva testing, or visual inspection for injection stigmata to verify eligibility [34-42]. These methods, of course, are not possible during online screenings, and using UDT or in-person/virtual consultation to confirm eligibility in Web-based studies would be time- and resource-intensive and could offset the advantages of online research.

Similarly, methods to recruit participants from specific geographic areas and verify residence in those areas can be difficult to implement. Even if study advertisements are targeted (ie, via local outreach and posting local flyers), once a survey link is revealed online and/or the link is shared with peers, people who live outside the target area can access the link. Many online survey platforms have the capacity to record geolocation based on user internet protocol (IP) address and allow researchers to restrict access to online surveys based on geolocation [43-45]. However, geolocation linked to IP address can be inaccurate because when a device is connected to a virtual private network or network address translation, only an external IP address is displayed, causing all devices to have identical IP addresses and geolocation. In addition, smartphones can display multiple different IP addresses within minutes because of network proxies within the carrier’s network, resulting in inaccurate geolocation based on IP addresses [46-48]. Recent research among MSM in Kentucky revealed that a substantial proportion of entries with ineligible geolocations based on IP addresses belonged to verified eligible participants [49].

An alternative to in-person UDT and/or visual inspection of participants to verify substance use and IP address geolocation to verify residential location is assessing knowledge about drug use and the target study area. Previous studies have used trained interviewers to assess prospective participants’ knowledge about the preparation of drugs for injection, administration of injections, and the size and color of needles and syringes, in addition to visual inspection for injection stigmata [42,50]. Other studies that include PWUD through noninjection routes have used interviewer-administered questionnaires to assess knowledge of street terminology, major formula doses (eg, milligram), and pill images [41,51]. To our knowledge, research that verifies residential eligibility through the assessment of knowledge about the target study area has not been conducted. However, assessing knowledge of local community features such as the names of nearby cities and towns, local businesses, and physical landmarks, in addition to in-person or targeted recruitment strategies (ie, direct marketing, respondent-driven sampling, and venue-based sampling) could be useful in enrollment of participants from a specified geographic area. Thus, substance use and local community *quizzes* could prove valuable in Web-based survey research among PWUD and in research targeting specific geographic areas.

### Objectives

The aim of this study was to explore the utility of using an online survey to screen and enroll young adult PWUD from rural Kentucky into an online survey about substance misuse and related risk behaviors. This study describes the implementation of an online eligibility screening quiz about the local community to verify residence in the target study area along with drug dose, appearance, and price to verify substance use.

## Methods

### Overview

Young adults who use opioids were recruited from August 2017 to July 2018 from 5 counties in rural Appalachian Kentucky to participate in an online survey, programmed in SurveyGizmo [42]. The survey contained questions about participants’ substance use, sexual and drug-related risk behaviors, and risk environments. Eligibility criteria included being 18 to 35 years old, living in the 5-county study area, and using opioids to get high in the past 30 days. Opioids included prescription pain pills, heroin, buprenorphine, methadone, and synthetic opioids. The study was funded to focus on individuals aged 18 to 35 years because of the disproportionate burden of opioid use and related harms (ie, hepatitis C and overdose) impacting young adults in rural settings [21-24,28,52].

Participants were recruited using both targeted and Web-based peer referral methods. Targeted outreach included distributing flyers at local businesses and organizations where young PWUD may be present (eg, tobacco shops, laundromats, gas stations, and social service offices), referrals from staff from another study on PWUD in the target area, and hosting community cookouts that advertised the study. Those who were eligible and completed the survey also had the option to refer peers through emailed or text messaged electronic peer referral coupons. Participants received US $10 for up to 3 eligible referrals who completed the survey. Study flyers and recruitment coupons had a URL for a study website hosted by WordPress [53], which had the link to the SurveyGizmo screening survey. The website also provided information for completing the screening assessment, informed consent, and survey. Flyers’ text included a university and study logo and stated that participants who lived in the 5-county study area were needed for a study on rural health. The flyers did not disclose that the study was focused on drug use.

Informed consent was self-administered for both the online screening and the survey. The consent also informed participants that UDTs would be administered to a random subsample of participants. To demonstrate comprehension, participants were required to answer 4 questions correctly at the end of the consent form that covered the content of the informed consent. After informed consent, participants were asked how they would like to be compensated. Options included cash, money wire, gift card, or an e-gift card of US $30. UDT compensation was US $25 and was given in-person at the time of urine specimen collection.

Before beginning the full online survey, eligibility was assessed using the date of birth to capture age and quizzes that examined knowledge about opioids and the local community to verify substance use and residence, respectively. Before initiating recruitment, we conducted pilot tests of these quizzes with young adult PWUD living in the study area. Information gathered was used to make adjustments to the quizzes to maximize utility and clarity before participant enrollment.

### Quiz to Verify Substance Use Eligibility

To verify substance use, people were asked questions about their use, followed by a quiz. First, people were asked to select all substances they had used to get high in the past 30 days from a list containing several opioids (eg, heroin, synthetic opioids, buprenorphine, methadone, and prescription opioids), nonopioids (eg, prescription sedatives or tranquilizers, cocaine, crack, methamphetamine, gabapentin, bath salts, and hallucinogens), and *other*, followed by a write-in response. Participants also had the option to select *none of these*. Those who had not used any substance to get high in the past 30 days were not quizzed and skipped the remaining substance use items.

People who reported using prescription opioids to get high were asked to specify which prescription opioid(s) they used using a checklist. People who reported using any opioid were then asked to specify which opioid they had used *most often* to get high in the past 30 days. Those who reported and specified which prescription opioid they had used were given the option to select that particular drug.

The drug quiz queried the opioid they reported using most often in the past 30 days. If they had used other nonopioids to get high, in addition to opioids, the *most often* follow-up question only listed opioids to ensure they were quizzed on a drug that related to eligibility criteria. Drug-specific opioid and nonopioid quizzes are described below.

#### Heroin Quiz

People who reported heroin as their most frequently used opioid in the past 30 days were administered a similar, 4-question quiz. First, they were asked what the most common size for a cap, or *one hit*, of heroin was in their county with the following response options: one-tenth of a gram (correct), one gram, five grams, and 20 grams. People were then asked how much 1 cap or *hit* of heroin cost in their town, with the following response options: US $0-$10, $10-$50 (correct), $50-75, and more than $75. Local law enforcement experts who arrange undercover drug purchases and local PWUD were consulted for information on *cap* size (1/10th of a gram or 100 mg) and heroin price (US $20 to $40 per cap).

Finally, people were asked to identify which photograph looked most like the heroin they buy in their county and were given 10 images as options, with 5 showing different types of powder heroin ranging from white to dark brown and 5 showing images that had textures and/or colors that would obviously not be heroin to a heroin user. People were required to get either the most common size or the cost for 1 cap of heroin correct to *pass* the heroin quiz. Image recognition was not included in the heroin quiz score, as it is possible that some people may only see heroin after it has been dissolved and heated for injection.

#### Prescription Opioid Quiz

**Figure 1:**
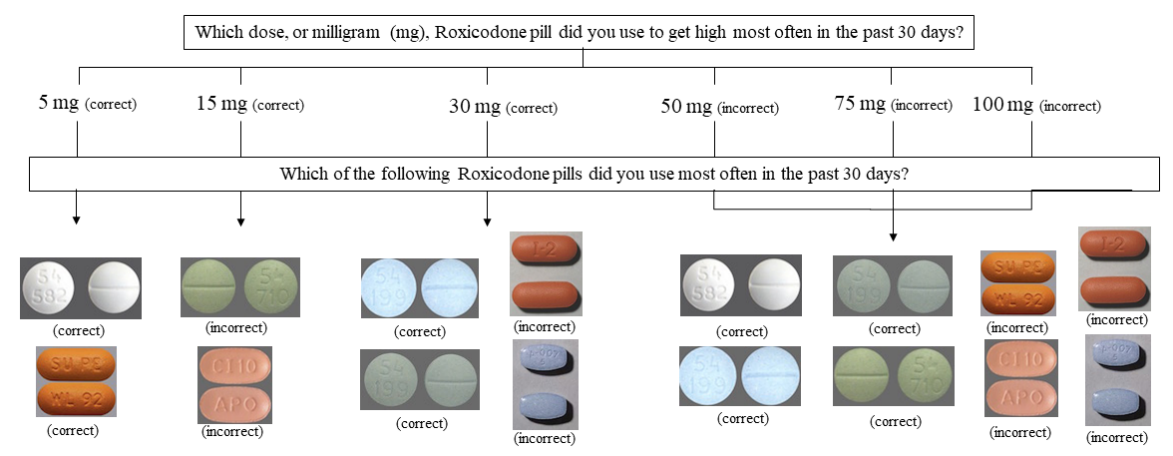
Example sequence of questions from the online screening eligibility process for prescription opioid quiz for Roxicodone.

For nonmedical use of prescription opioids, buprenorphine, and methadone, quizzes involved multiple-choice questions about dose (ie, choosing the dose from a list of real and fake milligrams options) and appearance (ie, recognizing an image from a set of correct and incorrect images). Because most prescription opioid pills, lozenges, films, or tablets are made in multiple doses and have different appearances depending on dose, formulation, and manufacturer, the quiz’s branching and skip patterns had to account for each drug/dose combination. Questions had a varying number of response options depending on the number of actual doses and images that were possible, such that 50% of options were correct and 50% were incorrect. For example, as shown in Figure 1, Roxicodone is manufactured as 5, 15, and 30 mg pills; therefore, the dose question had *6* response options so that 50% of options were correct and 50% were incorrect. Similarly, sets of response options for questions on Roxicodone images for each dose contained 50% correct responses. Incorrect dosage selection branched to an image question that provided images of all doses so that even if participants selected the incorrect dose, they still had the opportunity to identify the correct image.

People were also asked about the street price in an open-ended question. They were instructed to leave the answer blank if they did not know it. Because the study team was unable to gather information on street price for every possible prescription opioid, milligram, formulation, and manufacturer, this question was not included in the quiz score. People were only required to get either dose or image correct to *pass* the prescription opioid quiz.

#### Synthetic Opioid Quiz

Because of the recent emergence and rapidly evolving nature of the synthetic opioid market, we could not program a scorable quiz for synthetic opioids. If a person reported using synthetic opioids, they were asked where people normally get synthetic opioids (online, gas stations, drug paraphernalia stores, or other), what type of substance synthetic opioids were (pills, powder, liquid, and other), and price for 1 dose. The answers were not scored, and people who reported synthetic opioids as their most commonly used drug were automatically deemed eligible for the survey if they completed the screener and were otherwise eligible.

#### Nonopioid Quiz

People who did not report opioid use received a brief quiz because if quizzes were only given to those who initially reported opioid use, it could serve as a clue to people about what type of drug use would qualify them for the study. Therefore, to avoid unmasking eligibility criteria, people who had not used an opioid (ie, methamphetamine, cocaine, bath salts, or hallucinogens) were administered a 3-question quiz on the nonopioid drug they reported using in the past 30 days. The quiz asked about the drug’s color and texture (ie, pill, liquid, powder, rocks, or other), as well as an open-ended item on dose or common size for a bag of the drug. The nonopioid quizzes were not programmed to be scored.

### Quiz to Verify Residential Eligibility

To verify residence in the 5-county area, people were asked which state and county they had slept in most often in the past 6 months and then were administered a 5-question quiz assessing their knowledge of that county. Quiz items for study area counties were drawn at random from a 10-question bank developed specifically for that county. A question bank was used so that if a person tried to coach another respondent as they took it simultaneously or at a later date or tried to take the screening survey multiple times until they passed, it would be harder to share and/or learn the answers.

Quiz items contained yes/no and multiple-choice questions with the latter having 4 or 5 response options. For each eligible county, county-specific quiz items queried topics that were known widely within the county but most likely were unknown to people who did not live in the county. A total of 6 of the 10 quiz item topics were the same across counties; however, the remaining 4 items in the bank varied across eligible counties based on what features were applicable and salient (see Multimedia Appendix 1). Questions and topics were chosen based on suggestions from community members who attended local recruitment events and from local community partners. People were required to get 3 out of 5 questions correct to *pass* the county quiz.

A generic county quiz was administered when individuals reported living outside the 5-county study area to help mask which counties were eligible. The generic county quiz was pulled from a 10-question bank that had similar content presented in Multimedia Appendix 1. Responses to these items were not scored, as correct answers for all possible noneligible counties were unknown and simply stating that they lived outside the 5-county area disqualified persons from the study.

### Urine Drug Testing

A subsample (n=34) of survey participants who reported using opioids in the 24 hours before completing the screening were randomly selected to complete a 13-Panel iCup Drug Test within 3 days of the survey based on drug detection windows. The survey tool was programmed to randomly select participants for UDT if they completed the survey and reported using opioids in the past 24 hours. The iCup test is an extensive UDT for 13 different drugs including opiates (heroin and morphine), buprenorphine (Suboxone, Subutex, and Temgesic), methadone (Dolophine, Methadose, and Physetone), oxycodone (Percocet, Percodan, OxyContin, and Tylox), and propoxyphene (Darvocet and Darvon), as well as various stimulants, sedatives, and other drugs [54]. According to the manufacturer, most drugs appear in urine 2 to 5 hours after use, and drug detection windows vary based on several factors including frequency of use, route of administration, body mass, and age. Participants who were randomized to receive a UDT were contacted by the study staff to schedule an appointment at a local venue to obtain the urine specimen.

### Analysis

Descriptive statistics were used to describe results of the online eligibility screening algorithm and quizzes and to compare UDT results with self-reported opioid use. Nonparametric tests (eg, Kruskal-Wallis test and Spearman rank-order correlation) were performed because of non-normal distribution of outcome variables to explore associations across subgroups using SPSS Statistics version 25.0 (SPSS Inc).

### Ethics

All study procedures were approved by the University of Kentucky Institutional Review Board, and data were protected by a Federal Certificate of Confidentiality. To ensure anonymity, IP address and geolocation of the device used when completing the survey were not collected. All data were password-protected and stored on a secure server.

## Results

### Overview

**Figure 2:**
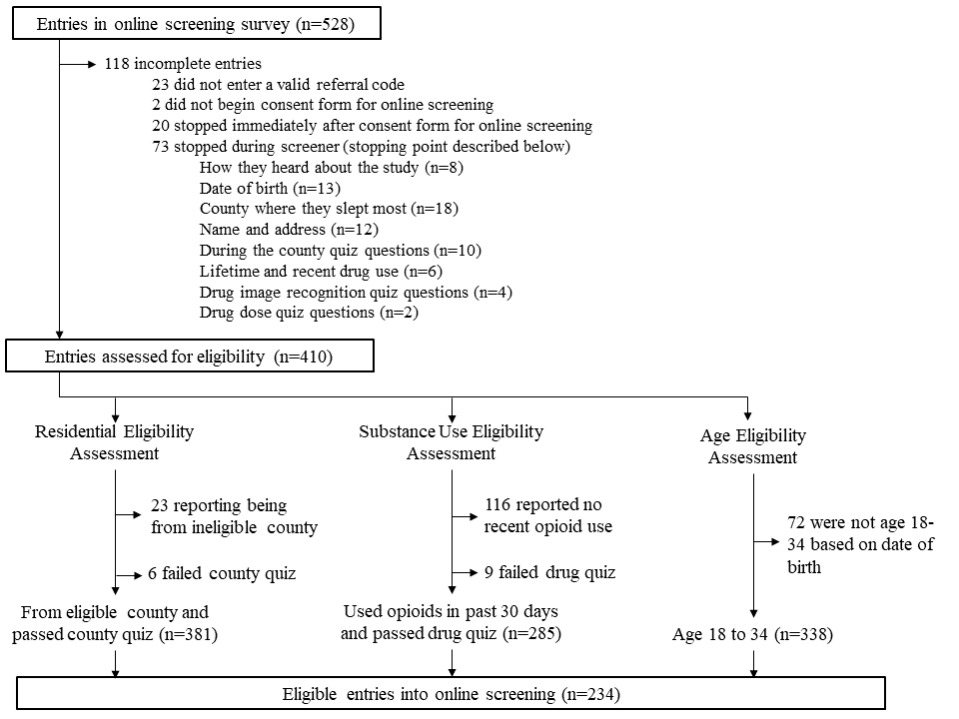
Outcomes of the online screening process from the online study of young people who use opioids.

Figure 2 describes the results of the eligibility screening. In total, there were 528 entries in the online eligibility screening survey, 22.3% (118/528) of which were incomplete (see Figure 2). Among the complete entries, the median time required to complete the screening was 6.14 min (interquartile range: 3.65-8.82 min). Of the 410 complete entries, 57.1% (234/410) were deemed eligible. Over half (229/410, 55.9%) were male, and the average age was 30 years (SD 11 years). Data integrity (ie, fraud detection) and final survey sample characteristics of eligible participants are published elsewhere [53]. Most (116/176, 65.9%) of the ineligible entries were due to not reporting any recent opioid use, followed by 41% (72/176) who reported being outside the eligible age range (18-35 years) and 13.1% (23/176) reporting living outside the study area. Only 6.8% (12/176) of ineligible entries were classified as ineligible because of failing the county quiz (n=5) and/or drug quiz (n=7). It should be noted that ineligibility data presented in Figure 2 are not exclusive; people may have been ineligible based on multiple criteria.

### Drug Quiz Results

In total, only 4 people failed the drug quiz for heroin, as a result of answering both the cap size and street price question incorrectly. Among the 168 people who passed the drug quiz for heroin, 167 (99.4%) recognized the image of powdered heroin, 159 (94.6%) answered the cap size question correctly, and 163 (97.0%) answered the street price question correctly. In total, 91.7% (154/168) answered the cap size and street price questions correctly. Because of the low variance on the quiz scores, we lacked the statistical power to examine variables associated with performance on the heroin quiz.

The most common prescription opioid that participants had used most frequently in the past 30 days and were, therefore, quizzed on was Percocet (n=18), followed by Norco (n=5), fentanyl (n=4), Roxicodone and Lortab (each with n=3), Tramadol and Tylox (each with n=2), and Opana and OxyContin (each with n=1). A total of 2 participants failed the drug quiz for prescription opioids, 1 for Percocet and 1 for Tylox. Among the 38 people who passed the drug quiz for a prescription opioid, 36 (95%) answered the dose question correctly, and 31 (82%) selected the correct image. Of those who selected incorrect prescription opioid images, 1 was for Norco and 6 were for Percocet. The 2 incorrect dose responses were also for the Percocet drug quiz.

In total, 74 people reported buprenorphine as their most frequently used drug in the past 30 days, with most (n=66) using buprenorphine pills. Among those quizzed about buprenorphine pills, 3 answered the dose and image questions incorrectly and failed the drug quiz. Of those who passed the drug quiz, 95% (60/63) answered the dose question and 92% (58/63) answered the image question correctly. In total, 84% (53/63) answered the dose and image questions correctly. Among those quizzed about buprenorphine strips, all passed the drug quiz, with 89% (8/9) answering both dose and image questions correctly.

A total of 4 people were quizzed on methadone, 1 on liquid methadone, and 3 on pills. No participant failed the methadone drug quiz. Only 1 person answered a methadone quiz question incorrectly, which was the methadone pill dose question.

### County Quiz Results

A Kruskal-Wallis test was conducted to examine differences in the quiz score across the 5 counties in the study area. No statistically significant differences in quiz scores were found across the 5 counties (χ_4_^2^=6.9, *P*=.14). Of the 6 parallel questions that were asked for every county in the study area (see Multimedia Appendix 1), the item querying smallest communities in the county was answered incorrectly most frequently, followed by if there was a jail or prison in the county, the local physical landmark specific to the county, and if there was a Walmart in the county. Among the 381 people who passed the county quiz for an eligible county, the average score was 4.9 (range 0-5) and most (91.1%) answered all 5 questions correctly.

Spearman rank-order correlation was used to investigate whether there was an association between length of residence in the county and county quiz score among those who were eligible and completed the survey. There was no statistically significant association between length of residence in the county and county quiz score (*r*_s_=.03, *P*=.74). Of note, length of residence could not be assessed as a correlate to county quiz score among those who failed the county quiz because duration of residence was only collected from those who participated in the study.

### Urine Drug Testing Results

Among the 44 individuals who were randomized to UDT, 34 completed the UDT within 3 days of their survey. Of these, 74% (25/34) tested positive for any opioid, including 27% (9/34) who tested positive for opiates (ie, heroin), 50% (17/34) who tested positive for buprenorphine, and 3% (1/34) who tested positive for oxycodone (see Table 1). Overall, 64.7% (22/34) tested positive for any stimulant. In addition, 6% (2/34) tested positive for benzodiazepine and 3% (1/34) for phencyclidine. Only 2 participants did not test positive for any drug.

Table 2 describes test results based on the opioid they reported (1) using most frequently in the past 30 days and (2) using in the past 24 hours. Slightly more than 50% (18/34) had urinalysis results that matched the opioid they reported using most frequently in the past 24 hours.

**Table 1:** Comparison of self-reported use of opioids with results of the 13-panel iCup urine drug test completed within 3 days of self-reported use.

Opioid used within past 24 hours^a^	Those with positive UDT^b^, n (%)	Days since survey completion, average (range)^c^	UDT detection window, days [54]	Detection threshold, ng/mL [54]
Heroin (n^d^=21)	8 (38)	0.22 (0-1)	2-4	2000
Buprenorphine (n^d^=9)	8 (89)	0.52 (0-3)	2-3	10
Percocet (oxycodone; n^d^=4)	1 (25)	0.75 (0-1)	2-4	100

^a^Participants had self-reported the opioid they used most often in the past 30 days and also reported use in the 24 hours before completing the screening.

^b^UDT: urine drug test.

^c^Days elapsed between survey completion and UDT completion. Zero means that the screening was performed the same day.

^d^The number of people who completed the UDT.

**Table 2:** Urine drug testing results of 13-panel iCup urine drug test completed within 3 days of self-reported use (N=34).

Drug		Positive UDT^a^, n (%)	UDT detection window, days [54]	Detection threshold, ng/mL [54]
Marijuana		18 (53)	15-30	50
**Opioids**		25 (74)	—^b^	—
	Opiates (heroin, morphine)	9 (27)	2-4	2000
	Methadone	0 (0)	3-5	300
	Oxycodone	1 (3)	2-4	100
	Propoxyphene	0 (0)	1-2	300
	Buprenorphine	17 (50)	2-3	10
**Stimulants**		22 (65)	—	—
	Methamphetamine	21 (62)	3-5	1000
	Amphetamine	21 (62)	2-4	1000
	Cocaine	3 (9)	2-4	300
Benzodiazepines		2 (6)	3-7	300
Phencyclidine		1 (3)	7-14	25
Barbiturates		0 (0)	4-7	300
Any drug (excluding marijuana and tricyclic antidepressant)		30 (83)	—	—

^a^UDT: urine drug test.

^b^Not applicable.

## Discussion

### Principal Findings

Web-based recruitment and data collection can be leveraged for research among hidden populations. However, tools for verifying behavioral and geographic eligibility criteria, such as recent substance use and residence, in Web-based research are lacking. This study demonstrated the utility of using online eligibility screening quizzes to verify substance use and residence in an online survey of young adult PWUD from rural Kentucky. In a random sample of participants who completed UDT within 3 days of their online screening, 73.5% (25/34) tested positive for an opioid, with drug detection windows ranging from 1 to 5 days. Only 2 did not test positive for any drug. In addition, most of those who reported recent opioid use (285/294, 97.0%) and living in the 5-county study area (381/387, 98.4%) were able to pass the respective quizzes.

Quizzes to verify substance use queried drug dose, price, and image recognition. People were able to offer accurate answers to these questions across opioid types. For example, among those who reported heroin as their most frequently used drug in the past 30 days, most (154/168, 91.7%) answered the cap size and street price questions correctly, and every participant except 1 was able to successfully recognize an image of powdered heroin. Most participants who passed a prescription opioid drug quiz were able to correctly answer dose and image questions (28/39, 71.8%). Of note, Percocet was the most common prescription opioid that participants reported using *most frequently* in the past 30 days and had a greater proportion of incorrect pill image and dose responses. During the study, researchers anecdotally discovered that the street name for Roxicodone (eg, Perc 30s) may have led people who were using Roxicodone to incorrectly select Percocet and subsequently be unable to correctly identify dose and pill images. Formula doses, pill images, and drug street names may vary across settings and should be considered when developing similar tools. PWUD within the setting may be a vital resource to ensure proper tool development. In addition, to avoid unmasking the eligibility criteria in studies focused on specific drug classes (such as opioids as done in this study), it is important to administer quiz questions for all drug classes.

People also performed well on quizzes that were used to verify their residence in the 5-county study area. Of those who reported being from an eligible county, 1.6% (6/387)failed the eligibility quiz. Questions that had the highest proportion of correct answers were those about businesses or facilities in the county (ie, whether there was a particular grocery store such as Kroger, Walmart, or local chains in their county, a particular pizza restaurant in their county). Questions that appeared to be most difficult for participants were multiple-choice items that asked them to identify the county’s largest town/city and which among the 5 lists of small communities was located in their county. Furthermore, depending on the length of a study and turnover in communities, changes in local businesses and facilities may need to be considered when utilizing community-specific quizzes.

Community member input and pilot testing were essential to the development of the county quizzes. In this study, community partners who worked in local public health and social service agencies helped develop sets of 10 questions for each of the 5 eligible counties; quizzes were then piloted with local PWUD. Initially, quizzes contained questions on high school mascots and colors, but feedback from local PWUD revealed that those who moved to the area more recently may not know information about local schools, so the questions were revised. Pilot-testing results highlight the need to engage community members in the development of quizzes and to pilot-test quizzes in the target sample. These findings may also reveal the need for future studies to collect data on duration of residence in the screening instrument and to potentially vary the threshold for passing based on the length of residence. In addition, to minimize inapposite participation that could result from advertising online or, more broadly, where geographic ineligibility could be more problematic, study advertising should be targeted. In this study, community cookouts, flyers, and outreach by local study staff were used to advertise the study, which may have resulted in the low percentage (23/410, 5.6%) of screening survey participants who reported being from a county outside the eligible study area.

### Limitations

Although using Web-based methods to screen and enroll young adult PWUD was successful in this study, there were limitations. Quizzes are limited in their ability to distinguish people who have ever used substances from those who have used recently, given that drug prices, doses, and appearances may not vary drastically over time. In addition, creating quizzes for nonprescription opioids such as heroin and synthetic opioids is more difficult because of lack of manufactured doses and potential inconsistencies in appearance. Quizzes for these drugs may, therefore, need to be more vague and consequently easier to pass. This study’s small strata-specific sample sizes (ie, by drug and by county) limited its ability to detect correlates to quiz performance and precluded a more rigorous statistical comparison of quiz results with urine drug screen outcomes. Future research could examine differences in quiz performance by drug type and examine other correlates to quiz performance. Other analytic approaches such as factor analysis were not employed in this study because each quiz was slightly different across counties based on county features and for each drug based on dosages, pill manufacturers, and appearances. In future research involving a larger, more homogenous sample (ie, in 1 interested in use of a single drug) or more limited geographic area, psychometric properties of quizzes could be evaluated.

Narrow UDT detection windows limited our ability to compare test results with self-reported past 30-day substance use [54]. Future research could utilize hair, saliva, or blood tests that have longer detection windows and better capture eligibility recall periods [37]. Finally, technological issues could also create a barrier to participation or lead to inaccurate ineligibility. Informal conversations with participants revealed that some had difficulty entering their date of birth because of the appearance of the question on some smartphone devices; others experienced problems with loading image questions because of internet speed and connectivity. Of note, 48.3% (72/151) of participants reported completing the survey on a smartphone, 28.9% (43/151) on a computer, 10.7% (16/151) on a tablet, and 12.1% (18/151) on some other device [55].

### Clinical and Research Applicability

Innovative screening approaches are becoming increasingly important with the rise in Web-based research. Methods that utilize technology are, therefore, necessary both to ensure enrollment of truly eligible participants and to prevent fraudulent participation. Online quizzes to screen and enroll PWUD hold promise for future research as an alternative to more time- and resource-intensive approaches that could offset the advantages of Web-based methods. Online eligibility quizzes could also prove useful for studies that are not Web-based, as they could be used for eligibility screening and thereby reduce burden on staff of screening study participants through interviewer-administered approaches. Furthermore, as technology evolves, new methods for eligibility verification may emerge, particularly in studies where participants are using smartphones. For example, image recognition software could be used to recognize injection stigmata in studies of people who inject drugs or to verify residence in a target community through photographs of landmarks. With advances in drug testing and remote diagnostic confirmation using smartphones through saliva- [56,57], urine- [57,58], and serum-based assays [57], smartphone-based testing also may be integrated into future online studies of PWUD. Geocaching [58] and global positioning system targeting technology such as those used in online gaming and gambling to validate that a patron is within authorized jurisdictional boundaries [59,60] may be used in the future to verify residential eligibility. Although new technologies are promising, until they are seamlessly integrated into survey platforms, strict data security measures are in place, and smartphone ownership is ubiquitous, quizzes to assess eligibility will continue to be an important tool for screening and enrollment of participants into online research.

## Appendix

Multimedia Appendix 1 Quiz questions used to verify residential eligibility in a 5-county study area.

## Abbreviations

ARC: Appalachian Regional Commission
CDC: Centers for Disease Control and Prevention
IP: internet protocol
MSM: men who have sex with men
PI: principal investigator
PWUD: people who use drugs
SAMHSA: Substance Abuse and Mental Health Services Administration
UDT: Urine Drug Testing
