# Supplementary material for: Web-Based Eligibility Quizzes to Verify Opioid Use and County Residence Among Rural Young Adults: Eligibility Screening Results from a Feasibility Study
Source: JMIR Res Protoc. 2019 Jun 18;8(6):e12984. doi: 10.2196/12984 (PMC6604504; doi:10.2196/12984)
Supplement: Multimedia Appendix 1 [file resprot_v8i6e12984_app1.pdf]

**Questions included on county quizzes**	**Number of county quizzes that contained item (out of five counties)**	**Number of people who received question [a]**	**Number of people who answered correctly (%)**
Is there a jail or prison in [county]?	5	222	217 (97.7)
Is there a Walmart in [county]?	5	196	195 (99.5)
Is there a Kroger in [county]?	5	176	176 (100.0)
Which of the following lists small communities in [county]?	5	199	189 (95.0)
What is the biggest city/town in [county]?	5	194	187 (96.4)
What is [local physical landmark specific to county]?	5	189	185 (97.9)
Which of the following [parks/physical landmarks] is in [county]?	4	279	272 (97.5)
Is there a McDonald's in [county]?	4	168	165 (98.2)
What is the name of the pizza restaurant downtown?	2	26	26 (100.0)
What of the following festivals is in [county]?	2	25	25 (100.0)
What river runs through [county]?	2	56	54 (96.4)
What is the local university's mascot?	1	109	107 (98.2)
What are the local university's colors?	1	95	90 (94.7)
What interstate runs through [county]?	1	54	54 (100.0)
They are rebuilding businesses downtown that were destroyed a few years ago by a [local natural disaster specific to county]?	1	13	12 (92.3)
Which of the following is the name of the [county] newspaper?	1	5	4 (80.0)
Which of these stores is in [county]?	1	12	12 (100.0)

a. The number of people who received each county quiz question varies because questions were randomly drawn from a question bank and the bank of questions varied across the five counties. Therefore, the same set of questions were not administered to every person who took the survey. In addition, there were varying numbers of respondents across counties.

Table 1. Quiz questions used to verify residential eligibility in a five-county study area
